# Prospective Clinical Testing of Regulatory Dendritic Cells in Organ Transplantation

**DOI:** 10.3389/fimmu.2016.00015

**Published:** 2016-01-28

**Authors:** Angus W. Thomson, Alan F. Zahorchak, Mohamed B. Ezzelarab, Lisa H. Butterfield, Fadi G. Lakkis, Diana M. Metes

**Affiliations:** ^1^Department of Surgery, Starzl Transplantation Institute, University of Pittsburgh School of Medicine, Pittsburgh, PA, USA; ^2^Department of Immunology, University of Pittsburgh School of Medicine, Pittsburgh, PA, USA; ^3^Department of Medicine, University of Pittsburgh School of Medicine, Pittsburgh, PA, USA

**Keywords:** dendritic cells, immune regulation, renal transplantation

## Abstract

Dendritic cells (DC) are rare, professional antigen-presenting cells with ability to induce or regulate alloimmune responses. Regulatory DC (DCreg) with potential to down-modulate acute and chronic inflammatory conditions that occur in organ transplantation can be generated *in vitro* under a variety of conditions. Here, we provide a rationale for evaluation of DCreg therapy in clinical organ transplantation with the goal of promoting sustained, donor-specific hyporesponsiveness, while lowering the incidence and severity of rejection and reducing patients’ dependence on anti-rejection drugs. Generation of donor- or recipient-derived DCreg that suppress T cell responses and prolong transplant survival in rodents or non-human primates has been well-described. Recently, good manufacturing practice (GMP)-grade DCreg have been produced at our Institution for prospective use in human organ transplantation. We briefly review experience of regulatory immune therapy in organ transplantation and describe our experience generating and characterizing human monocyte-derived DCreg. We propose a phase I/II safety study in which the influence of donor-derived DCreg combined with conventional immunosuppression on subclinical and clinical rejection and host alloimmune responses will be examined in detail.

## Introduction

While rates of acute renal transplant rejection have improved dramatically since the advent of calcineurin inhibition (CNI) >30 years ago, similar improvement in long-term graft survival has not been achieved. This reflects the inability of conventional immunosuppressive agents to prevent late graft dysfunction leading to transplant failure (1, 2). Moreover, conventional immunosuppression is associated with significant morbidity and mortality due to cardiovascular, infectious, and pro-neoplastic side effects. Attempts to improve long-term survival, while reducing the burden of immunosuppression, have not been particularly fruitful to date. While the recent introduction of co-stimulation blockade, although renal-sparing, has resulted in an increased incidence of acute rejection (3), use of depleting antibody (Ab) as induction therapy at the time of transplantation has also failed to guarantee safe withdrawal of CNI, even in patients with stable graft function (4, 5). Furthermore, efforts to induce donor-specific tolerance using hematopoietic stem cell transplantation, an approach first shown to be successful many years ago in mice (6), have yielded promising results, but many hurdles remain in terms of safety and widespread applicability (7).

Our long-term goal is to develop a novel, safe, donor-specific induction (pre-conditioning) approach that will promote sustained, donor-specific immune hyporesponsiveness, while lowering the incidence and severity of acute and chronic rejection and reducing patients’ dependence on anti-rejection drugs. There is recent evidence that, by exploiting inherent mechanisms of immune regulation, it may be possible to achieve this goal. Rare, naturally occurring regulatory immune cells, either innate [regulatory dendritic cells (DCreg)] or adaptive [regulatory T cells (Treg)], critically regulate immunity, can promote antigen (Ag)-specific T cell hyporesponsiveness, and prevent adverse immune reactions (self-tolerance) in the healthy steady-state (8, 9). Moreover, in small animals, the adoptive transfer of DCreg (10–13) or Treg (14) can prolong allograft survival and induce donor-specific tolerance to organ transplants (15). Other regulatory immune cells with potential therapeutic applications include regulatory macrophages [Mreg; (16–18)], myeloid-derived suppressor cells [MDSC; (19)], T regulatory type-1 cells [Tr1 cells; (20)], and regulatory B cells (21). In addition to *ex vivo*-expanded Treg, now entering phase I/II trials in organ transplantation^1^, a compelling rationale has emerged for clinical testing of DCreg, i.e., donor or recipient blood monocyte-derived DC generated and modified *ex vivo* to promote their inherent regulatory properties (13, 22–24). Thus, we and others have shown that, in rodents, infusion of DCreg of donor or recipient origin before or after transplantation, including their use in combination with conventional immunosuppressive agents, can promote indefinite organ allograft survival. More importantly and uniquely, using a robust, clinically relevant, non-human primate (NHP) model with minimal immunosuppression, we have shown that infusion of donor-derived DCreg, 1 week before transplant, safely prolongs major histocompatibility complex (MHC)-mismatched, life-sustaining renal allograft survival, with no evidence of host sensitization (25). Equally significant is our demonstration that this therapeutic effect is associated with selective attenuation of donor-reactive memory T cell (Tmem) responses (25, 26), an important barrier to improvement of long-term graft survival (27, 28).

We have now generated good manufacturing practice (GMP) grade human DCreg from elutriated peripheral blood monocytes and demonstrated both their stable resistance to maturation under inflammatory conditions and their ability to negatively regulate alloreactive T cell responses. We have also established release criteria for clinical testing and plan to conduct a safety trial of donor-derived DCreg in adult, *de novo*, live-donor renal transplantation. To our knowledge, this promising donor-specific induction approach to regulatory immune cell therapy in clinical organ transplantation is unique. It is distinct from the testing of recipient blood monocyte-derived DCreg in live-donor renal transplantation currently being conducted at the University of Nantes, France, as part of The ONE Study (29, 30).

## The Case for DCreg Therapy in Organ Transplantation

Extensive pre-clinical studies that we and others have conducted in rodents and human surrogate models provide compelling evidence of the potential of regulatory immune cell therapy to improve allograft outcomes and, in many instances, promote donor-specific tolerance (15). The case for testing DCreg generated *ex vivo* in human transplantation is particularly compelling (13, 23, 24) for the following reasons. First, DC are uniquely well-equipped, professional Ag-presenting cells (APC) that potently regulate innate and adaptive immunity (31, 32). Second, in many animal studies, DCreg adoptively transferred to graft recipients *before* transplant induce Ag-specific T cell unresponsiveness (13) and promote indefinite organ allograft survival. Moreover, this beneficial effect on graft survival does not appear to depend on the *in vivo* persistence of intact DCreg (33–35). Indeed, the apparent independence of efficacy and regulatory mechanisms on the persistence of intact donor DCreg may be a distinct advantage over other cell therapy approaches. Thus, e.g., Treg therapy may require costly repeated infusion of very large numbers of expanded cells (36, 37) and their sustained viability/replication may be required to achieve a therapeutic effect. *Third*, an important attribute of DCreg is their ability to regulate, in addition to *de novo*-primed effectors, preformed Tmem responses (38–40) that, either due to preformed memory to alloAgs or due to molecular mimicry and cross-reactivity with human leukocyte antigens (HLA) (41), represent a major barrier to long-term graft survival in humans (27, 28, 42, 43). *Fourth*, in normal humans, local adoptive transfer of monocyte-derived DCreg has been shown to induce Ag-specific unresponsiveness to nominal Ags (44, 45). *Fifth*, using minimal immunosuppression in a robust NHP model, we have reported that a single infusion (3.5−10 × 10^6^/kg) of donor-derived DCreg, 1 week before transplant, safely prolongs renal allograft survival, with no evidence of host sensitization (25). Importantly, this effect is associated with attenuation of donor-specific, alloreactive Tmem responses (25, 26).

The unique phase I/II trial of donor-derived DCreg that we now propose in live-donor renal transplantation is essentially a dose-escalation safety trial in which the cell product will be administered, once only, concomitant with mycophenolic acid (MPA), 1 week before transplantation to patients receiving standard immunosuppression (CNI, MPA, and steroids). Successful safety evaluation of our strategy and any evidence of inhibition of early, acute subclinical or clinical rejection, and/or attenuation of long-term anti-donor immunity would justify broader evaluation of DCreg efficacy in renal transplantation. This would potentially address unmet needs of CNI-free immunosuppression and/or realize the unmet goal of improving long-term allograft survival, without increasing the burden of immunosuppression.

Thus, in future studies, it would be of interest to evaluate the influence of DCreg combined with co-stimulation blockade (Co-B) to ascertain whether the incidence of rejection episodes encountered with Co-B (3) can be reduced. Furthermore, evidence of a beneficial effect of DCreg pre-conditioning in early clinical trials might justify evaluation of immunosuppressive drug curtailment. It is likely that the DCreg approach can be applied readily in the clinic since, based on pre-clinical testing, a single infusion of a relatively small number of DCreg is sufficient to achieve the salutary effect. Therefore, neither expensive expansion of the cell product, nor repeated infusion may be necessary. It is also probable that donor-derived DCreg will have broader clinical applications to encompass recipients of renal and other organ transplants from *deceased* donors. Indeed, rodent studies have shown that delaying DCreg infusion until 7 or 14 days post transplant is (still) effective in prolonging graft survival (46, 47), thus providing ample time to prepare DCreg from deceased donors.

## Novelty of the Approach

Several closely interrelated aspects of our proposed clinical trial of DCreg in live-donor renal transplantation are highly innovative. *First*, we have generated a highly-purified GMP cell product (allograft donor blood monocyte-derived DCreg) distinct from those [autologous tolerogenic DC (not pulsed with donor antigen), Treg, Type-1 regulatory T cells (Tr1) cells, Mreg, and mesenchymal stem cells] being investigated by other groups, which satisfies phenotypic and functional release criteria. The manufacturing process is relatively simple, comparatively short and highly reproducible. Second, while early pilot studies have begun to examine the safety of *autologous* DCreg in human autoimmune diseases (48–50) and organ transplantation (29), this will be the first study to test *allogeneic* (donor-derived) DCreg in human organ transplantation. *Third*, our proposed mechanistic studies will address our *hypothesis* that, in addition to inhibition of *de novo* T cell priming and memory reactivation against donor HLA Ags, DCreg infusion will selectively undermine early inflammation that fuels anti-donor effector/Tmem responses and promote specific T cell unresponsiveness to donor that we will monitor sequentially in blood and protocol biopsies. We will also generate novel insight into the persistence/longevity of donor-derived DCreg in graft recipients. Of particular relevance, based on our NHP transplant data, will be analyses of *de novo*-primed T cell and Tmem phenotype and function and the potential of establishing new biomarkers of donor-specific hyporesponsiveness based on the profile of donor-reactive T cells. *Fourth*, since protocol biopsies will be performed, we will gain preliminary insight into the influence of DCreg on the incidence of subclinical rejection, an important predictor of long-term graft outcomes by analyzing graft-infiltrating T lymphocytes. By contrast, traditional immunosuppression trials have focused on the incidence of clinically evident rejection as a principal endpoint.

## Rationale for Testing DCreg in Human Kidney Transplantation

Dendritic cells are highly specialized, bone marrow-derived APC [first described >40 year ago (51)] that induce or regulate innate and adaptive immunity (13, 32, 52–54). While DCreg play a crucial role in maintaining self-tolerance in the healthy steady-state (8, 55, 56) over the past 20 year, our research and others have revealed that these cells can subvert naïve T cell and Tmem responses by various mechanisms (13, 22, 57–59) and that DCreg can induce or restore T cell tolerance in animal models of autoimmune disease (60–63) or organ transplant rejection (12, 13, 22, 64). In experimental transplantation, both donor-derived allogeneic DCreg and donor Ag-pulsed host autologous DCreg are effective. Importantly, our work has also confirmed that adoptive transfer of donor-derived DCreg can safely regulate T cell responses in clinically relevant NHP models, including MHC mis-matched organ allograft recipients (25, 65), an important bridge to clinical testing. There is also well-documented evidence that adoptive transfer of DCreg (*in vitro*-generated autologous DC) via local administration can control T cell responses to model Ags (flu matrix peptide and keyhole limpet hemacyamin) in human healthy volunteers (44, 45). Important insights gained from *in vitro* studies and animal models have driven the recent development of clinical grade human DCreg (66–70), with the potential to treat autoimmune disease or enhance transplant survival, while reducing patients’ dependence on immunosuppressive drugs. Phase I safety trials, in which autologous DCreg have been administered locally, have been conducted in type-1 diabetes (48) and rheumatoid arthritis (RA) (49, 50), with results that emphasize the feasibility, safety, and potential efficacy of DCreg therapy.

Based on these findings, we hypothesize that DCreg infusion, as an adjunct to conventional immunosuppression, can improve long-term renal allograft and patient outcomes, with minimal early adverse events, by targeting both innate immunity and preformed memory responses. It also carries the prospect of enabling immunosuppression reduction in stable patients or converting to CNI-free immunosuppression, without increasing the incidence of rejection.

Our laboratory has had a major focus on the characterization and therapeutic efficacy of DCreg, especially in experimental pancreatic islet, skin, and organ transplantation (46, 64, 71–79). These studies include the first observations that these regulatory innate immune cells, deficient in MHC and co-stimulatory molecule expression and in the production of pro-inflammatory cytokines, could subvert alloAg-specific T cell responses, *in vitro* and *in vivo* (72, 80). In addition, we have extensive experience in the characterization and immune profiling of human T lymphocytes, including the contribution of naïve T cell and Tmem subsets to the alloimmune response, and the effects of induction therapy on regulatory T cell and Tmem subsets in relation to clinical outcome in kidney transplantation (41, 81).

## Evidence in Support of DCreg Therapy in Transplantation

We summarize below evidence from rodent, NHP, and human studies that support the safety and, in the case of pre-clinical models, the efficacy of DCreg in solid-organ transplantation.

### Rodent Observations

We and others have shown that combination of pre-transplant (day −7) infusion of donor-derived DCreg, either alone or with low doses of immunosuppressive agents, can induce donor-specific organ transplant tolerance in rodents (12, 74, 82–85). The route of administration, dosage, dosage regimen, and duration of dosing (single i.v. infusion of up to 5 × 10^6^ per kg of donor DCreg, 7 days prior to transplantation) that we propose in a phase I clinical trial are, therefore, supported by experiments in rodents [Table 1 and (12, 73, 74, 82–86)] and NHP (25). It is also important to note that use of conventional “standard of care” (SOC) immunosuppressive agents (MPA, CNI, or steroids), together with DCreg, promotes long-term allograft survival in rodents [Table 2 and (46, 84, 87, 88)]. This is of direct relevance to the use of SOC immunosuppressive therapy in our proposed clinical trial.

**Table 1:** **Promotion of indefinite heart or renal allograft survival in rodents by infusion of donor-derived DCreg**.

DC source	Species	DC culture conditions	Route of injection	When administered^a^	Additional host treatment	MST	Reference
MoDC	rat	GM-CSF	i.v.	Day + 14/15	None	>160 days	Hayamizu et al. (86)
BMDC	mouse	GM-CSF + TGFβ	i.v.	Day-7	Anti-CD40L mAb	>100 days (40%)	Lu et al. (73)
BMDC	mouse	Low GM-CSF	i.v.	Day-7	None	>100 days	Lutz et al. (12)
BMDC	mouse	GM-CSF + IL-4 + NF-κB ODN + Ad CTLA4Ig	i.v.	Day-7	None	>100 days (40%)	Bonham et al. (74)
BMDC	rat	GM-CSF + IL-4	i.v.	Day-7	ALS	>200 days (50%)	DePaz et al. (85)
BMDC	mouse	Low GM-CSF	i.v.	Day-7	Anti-CD54 mAb + CTLA4Ig	>100 days	Wang et al. (83)
BMDC	Rat^b^	GM-CSF + IL-4 + dexamethasone	i.v.	Day-10	CTLA4Ig + cyclosporine	>100 days	Mirenda et al. (84)

*^a^In relation to transplantation on d0*.

*^b^Renal transplant*.

*Ad, adenoviral vector; ALS, anti-lymphocyte serum; BMDC, bone marrow-derived dendritic cells; i.v., intravenous; MoDC, monocyte-derived DC; MST, mean graft survival time; ODN, oligodeoxynucleotides decoys*.

**Table 2:** **Evidence that use of standard-of-care immunosuppressive agents (corticosteroid, MMF, and CNI) together with DCreg promote long-term allograft survival in rodents**.

Agent	Type of allograft (species)	Reference
MMF	Pancreatic islet (mouse)	Adorini et al. (87)
Dexamathasone	Renal (rat)	Mirenda et al. (84)
Tacrolimus	Composite tissue (rat)	Eun et al. (88)
Cyclosporine	Composite tissue (rat)	Ikeguchi et al. (46)
	Renal (rat)	Mirenda et al. 2004 (84)

*MMF, mycophenolate mofetil; CNI, calcineurin inhibitor*.

### NHP Observations

**Figure 1:**
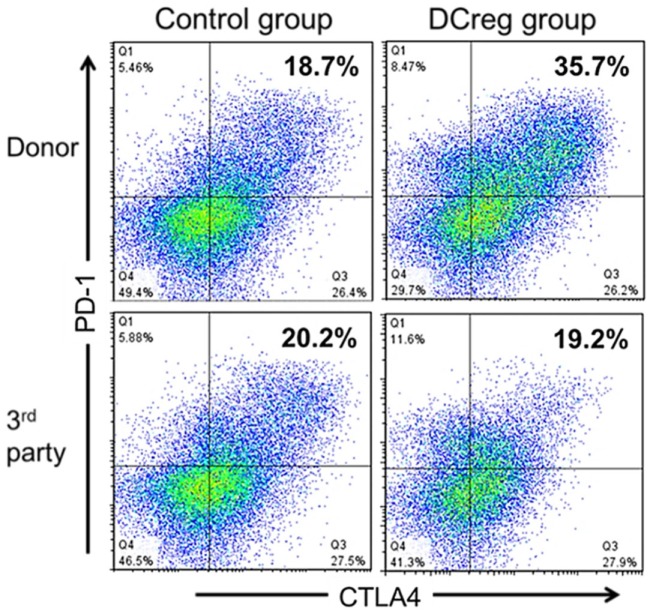
**DCreg infusion enhances programed death (PD)-1 and cytotoxic T lymphocyte antigen-4 (CTLA4) expression by donor-reactive CD4^+^Tmem in renal allograft recipient monkeys**. Incidences of PD1^+^ CTLA4^+^ populations in *ex vivo*-stimulated CD95^+^CD4^+^Tmem from representative control and DCreg-treated monkeys (*n* = 4 monkeys analyzed/group). Recipient PBMC obtained 28 days after transplantation, were co-cultured with either donor or third party stimulators (T cell-depleted PBMC) for 5 days before flow cytometric analysis. The enhanced incidence of PD1^+^CTLA4^+^Tmem in response to donor, but not third party stimulation suggests selective attenuation (exhaustion) of donor-reactive Tmem. According to Ezzelarab et al. (25), Figure 5.

Non-human primate transplant models are considered important predictors of the safety and efficacy of experimental immunosuppressive/tolerogenic regimens since the NHP immune system more closely resembles that of humans than mice, and since, as in humans (but not in mice), Tmem present an important and difficult to overcome barrier to induction of donor-specific tolerance (41, 89–91). We have used a robust, MHC-mismatched, life-sustaining rhesus macaque renal transplant model to evaluate the safety and efficacy of donor-derived DCreg therapy (25). In these studies, DCreg were generated from CD14 immunobead-isolated blood monocytes in a single leukapheresis product of the prospective kidney donor in granulocyte macrophage-colony-stimulating factor (GM-CSF) and IL-4. During the 7-day culture period, vitamin D3 (VitD3), a nuclear factor κβ inhibitor that impairs DC differentiation and maturation (92, 93) and IL-10, that converts immature DC into tolerogenic APC (94), were added to promote the maturation-resistant DCreg phenotype (95). We tested whether DCreg of donor origin, infused prospectively (once only) 7 days before transplant, could safely prolong graft survival using a minimal immunosuppressive regimen of co-stimulation blockade [CTLA4Ig = cytotoxic T lymphocyte Ag 4:Ig (abatacept)] and mechanistic target of rapamycin (mTOR) inhibition [rapamycin (sirolimus)]. Our findings (25) clearly show that (1) no adverse effects were encountered, (2) no evidence of host sensitization was detected, as determined by circulating anti-donor alloAb levels, (3) graft survival time was prolonged significantly (threefold increase) in the group given DCreg compared to those recipients that did not receive the cell infusion, (4) weight loss and proteinuria were less marked in DCreg-infused monkeys, and (5) evidence was obtained of significant, donor-specific attenuation (exhaustion) of Tmem responses as evidenced by upregulation of concomitant programed death (PD)-1 and CTLA4 expression (Figure 1), reduced memory:regulatory T cell ratios in peripheral blood, and reduced CD8^+^ effector T cell responses in the transplant (25, 26).

### Human Observations

Several pharmacologic agents and cytokines have been used to generate GMP grade autologous human DCreg for prospective clinical use in chronic inflammatory diseases, including type-1 diabetes, RA, and multiple sclerosis (48–50, 66–68). The safety of locally administered, autologous, monocyte-derived DCreg in type-1 diabetes or RA patients has been reported (48–50). To our knowledge, there has been no human experience with donor-derived DCreg in human organ transplantation. However, clinical experience with a closely related, donor-derived myeloid lineage cell product in either deceased- or live-donor renal transplantation is relevant to the proposed investigation of DCreg in organ transplantation. Thus, “immunoregulatory macrophages” (Mreg) or “transplant acceptance-inducing cells” have been investigated by Hutchinson and colleagues in Germany as immune-conditioning therapy in human renal transplantation (96). The phenotype of these cells identifies them as a subtype of partially mature macrophages (96). Initially, they were generated from deceased-donor splenic mononuclear cells cultured in macrophage (M)-CSF and IFNγ for 5 days and administered i.v. on post-transplant day 5. All patients (*n* = 12; with 3–5 total MHC-mis-matches) received ≥0.55 × 10^6^ viable Mreg/kg (range 0.55–7.52 × 10^6^/kg) and were immunosuppressed at the outset with tacrolimus, sirolimus, and glucocorticoids. They were then weaned from steroid therapy, if clinically appropriate, on day 28 post-transplant. Administration of comparatively large numbers of these cells (up to 5 × 10^8^ viable cells) via a central line was safe, with no evidence of graft-versus-host reactions induced by the Mreg or contaminating lymphocyte populations. Furthermore, as in our NHP DCreg studies, there was no evidence that human Mreg sensitized the recipients to donor Ags, or that the cells themselves could otherwise accelerate rejection. Importantly, none of the study participants experienced any delayed complications from Mreg infusion (mean follow-up time 36 months). Thus, it was concluded that the infusion of (donor-derived) Mreg was practicable and safe in the acute and medium term.

The same group of investigators have also infused donor-derived Mreg to live-donor kidney transplant recipients (*n* = 5), 5 days before renal transplantation (97). A larger number of Mreg and a different immunosuppressive regimen [anti-thymocyte globulin (ATG), tacrolimus, and steroids] were employed. PBMC were isolated from donor leukapheresis products 14 days before transplant. On day 9 pre-transplant, non-adherent PBMC from leukapheresis products of the prospective graft recipients were added (2.10^7^/ml) to the donor-derived Mreg and the co-cultures of donor origin Mreg and recipient PBMC maintained for a further 4 days until infused (1.74−10.39 × 10^7^ Mreg/kg) 5 days before transplant. No complications were observed. Moreover, there was no evidence that infusion of donor-derived Mreg prior to transplantation could sensitize recipients to donor Ags or otherwise accelerate graft rejection. As in the earlier study, it was concluded that preoperative treatment of live-donor kidney transplant recipients with Mreg was clinically practicable and safe in the acute and medium term.

In a further (2011) publication (98), the same group (plus additional authors) reported on two live-donor renal transplant patients who were given donor-derived Mreg (99) cultured for 6 days with M-CSF before stimulation with IFNγ for a further 24 h, and then administered 6 or 7 days before transplant. In this case, the Mreg were CD14^−/lo^, HLA-DR^+^, CD30^−/lo^, CD86^+^, CD16^−^, toll-like receptor (TLR)2^−^, and CD163^−/lo^. One patient (single HLA-B and HLA-DR mismatches) received 8.0 × 10^6^ cells/kg and the other (fully HLA-mismatched) received 7.1 × 10^6^ cells/kg. Labeling of a proportion of the infused Mreg with [^111^In]-oxine in one patient and whole-body single photon emission computed tomography imaging (SPECT) revealed that the Mreg located initially in the lungs, but after 2.5 h were evident in the circulation and had begun to accumulate in the liver and spleen. Twenty-four hours after Mreg infusion, signal from the lung had diminished substantially and the cells had accumulated in the liver, spleen, and bone marrow. Absence of signal from the patient’s urinary tract throughout the 30 h follow-up suggested that the majority of labeled infused cells remained alive. No unexpected adverse events were observed in either patient. At 3 and 2 year, respectively, post-transplant, the patients were taking once-daily or twice-weekly tacrolimus. Despite early minimization of immunosuppressive therapy, neither patient underwent an acute rejection episode during the 3-year follow-up period.

## Potential Mechanisms of the Long-Term Maintenance of Suppression after DCreg Administration

The *in vivo* mechanisms whereby infusion of donor (or recipient)-derived DCreg restrains alloimmunity and promotes long-term survival of experimental organ allografts are not well understood. In mice, there is evidence that donor-derived DCreg infused before transplantation are targeted by host NK cells and, thus, short-lived (35). They are reprocessed by quiescent host splenic DCs for presentation of alloAg to indirect pathway CD4^+^ T cells. This results in abortive activation and deletion of T effector cells without impairing the incidence of indirect CD4^+^ Foxp3^+^ Treg, thus enhancing the regulatory to effector T cell ratio (33, 100). It appears, therefore, that mechanisms that sustain long-term graft survival are not dependent on persistence of intact donor DCreg.

## Proposed Clinical Testing of DCreg in Renal Transplantation

Here, we propose a protocol for the generation and testing of donor-derived DCreg in a phase I clinical trial in renal transplant recipients receiving conventional immunosuppressive therapy.

### Generation, Purity, and Yield of hu DCreg

**Figure 2:**
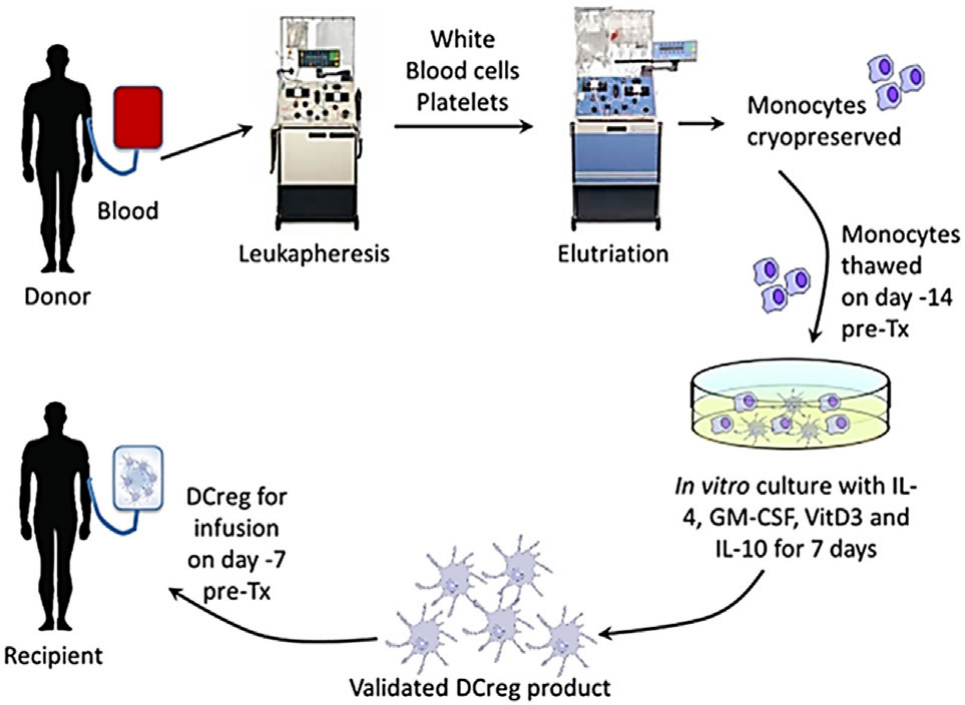
**Generation of DCreg from elutriated blood monocytes of the prospective renal allograft donor in GM-CSF, VitD3, and IL-10, and infusion of the validated cell product into the graft recipient 7 days before transplant**.

To ensure sufficient DCreg yields, blood monocytes will be obtained and banked in high purity by elutriation from cryopreserved leukapheresis products of the prospective transplant donors approximately 28–15 days before scheduled transplantation (Figure 2). Fourteen days before transplantation, monocytes will be thawed and DCreg generated for infusion into the prospective graft recipient on day-7 (Figure 1). In our experience, whole individual leukapheresis products from non-mobilized, healthy adult volunteers yield 4.3 ± 1.05 × 10^9^ PBMC. Recovery of monocytes post-elutriation [consistently ≥90% pure with <1% CD3^+^ T cell contamination (*n* = 4)] represents, on average, 25% of the total PBMC. The phenotype of the purified monocytes, determined by flow cytometry, is HLA-DR^+^ CD40^lo^ CD80^lo^ CD86^+^, programed death ligand (PD-L) 1^lo^, CD14^+^.

The DCreg are generated from thawed monocytes in serum-free Cell Genix (Cellgro) medium, supplemented with 5% certified human AB serum and recombinant human (rhu) GM-CSF (1000 units/ml) and rhu IL-4. These cytokines are added at the start of culture (day 0) and on day 4. VitD3 and rhu IL-10, which suppress DC maturation (94, 101, 102), are also added on day 4. The culture period is 7 days. We consistently generate sufficient, highly purified DCreg from elutriated peripheral blood monocytes (yield = 17 ± 7% of starting monocyte number) from a single whole leukapheresis product to administer up to 2.0−2.5 × 10^6^ per kg to a 70 kg recipient. To obtain larger numbers of DCreg for a higher dose, a second donor leukapheresis may be required.

The DCreg harvested at day 7 of culture are consistently >94% pure, with ≤0.1% contaminating CD3^+^T lymphocytes determined by flow cytometry. It is especially significant that the incidence of T cells is so low since these are the cells that are of concern regarding risk of graft-versus-host disease. The DCreg consistently exhibit an immature phenotype compared to control DC (i.e., immature DC generated in DC media without VitD3 and IL-10) and are HLA-DR^+^, CD11c^+^, CD14^−^, CD40^lo^, CD80^lo^, CD86^lo^, PD-L1^hi^, CCR7^+^, CD83^lo^. High expression of PD-L1(= B7-H1), a negative regulator of T cell responses and a consistently high PD-L1:CD86 ratio [determined as: PD-L1 mean fluorescence intensity (MFI) ÷ isotype control/CD86 (MFI) ÷ isotype control] conforms to that of DCreg with potential to subvert alloreactive T cell responses.

### Function of DCreg

Investigation of the function of hu DCreg harvested at 7 days of culture, including their responses to the TLR4 ligand bacterial lipopolysaccharide (LPS), CD40 ligation, or a pro-inflammatory cytokine cocktail, will ensure their regulatory properties. The DCreg we have generated display robust resistance to phenotypic and functional maturation in response to factors that promote the maturation of control DC, confirming that they are refractory to stimulation under inflammatory conditions. In particular, the inability of DCreg exposed to LPS to release pro-inflammatory and immunostimulatory cytokines (tumor necrosis factor [TNF]α and IL-12p70) is profoundly inhibited, while production of anti-inflammatory IL-10 is preserved, resulting in marked reversal of the IL-12:IL-10 and TNFα:IL-10 ratios.

The ability of the DCreg exposed to LPS to induce hyporesponsiveness of normal allogeneic T cells is of paramount importance. Therefore, DCreg (ratio 1 DCreg:40 T cells) should induce minimal CD4 and CD8 T cell proliferation, IFNγ and IL-17 release, or granzyme B production in the responder CD4^+^ T cells over 4 days in culture. This analysis provides further assurance of the inability of the DCreg, despite exposure to a potent pro-inflammatory stimulus (LPS), to stimulate allogeneic effector T cell responses. Similarly, marked attenuation of alloreactive CD8^+^ T cell responses is observed.

Thus, we contend that infusion of DCreg that are (1) phenotypically immature, (2) resistant to maturation under inflammatory conditions, and (3) able to induce allogeneic T cell hyporesponsiveness *in vitro* will not induce sensitization of prospective recipients following their adoptive *in vivo* cell transfer and rather, will induce donor-specific T cell hyporesponsiveness. Our plan to closely monitor study patients for evidence of development of donor-specific alloAb production and anti-donor T cell reactivity will allow detection of any increase in anti-donor immune effector activity in the unlikely event it should occur.

### Release Criteria for DCreg

The DCreg generated for infusion will undergo rigorous testing at specified time points during their manufacture from blood monocytes. The following release criteria will be considered as crucially important: DCreg yield (sufficient cells to allow infusion of the target number per kilogram), percent purity (>95% DC, <1% T cells), and viability (>70%); sterility; DCreg phenotype: phenotypic characterization will be performed by flow cytometry to monitor CD86 and PD-L1 expression before and after LPS stimulation, compared to conventional DC cultured in GM-CSF + IL-4 and not VitD3 and IL-10. High PD-L1 and low CD86 expression, before and after LPS stimulation, with a PD-L1:CD86 ratio >3.5 (based on pre-clinical results) will be used as a release criterion. The 3.5 ratio is based on many analyses in which a ratio of 3.5 or above was associated with a cytokine profile and T cell stimulatory profile consistent with the induction of alloreactive T cell hyporesponsiveness. DCreg function: supernatants from cultures (DCreg exposed or not to LPS) will be tested by ELISA to assess the lack of IL-12p70/TNF-α and the presence of IL-10 production, consistent with their regulatory properties and their resistance to maturation. We consider this, in addition to the tests above, a simple, reproducible, release criterion that can be applied before release of the DCreg product for infusion.

### DCreg Infusion

We plan to test three dose levels of DCreg in three separate groups of recipients (*n* = 5/group, with 4 receiving DCreg and 1 “control” subject receiving concomitant pre- and post-transplant immunosuppression without DCreg): dose 1: 0.5 × 10^6^ cells/kg body weight; dose 2: 2.5 × 10^6^ cells/kg body weight; dose 3: 5 × 10^6^ cells/kg body weight.

### Concurrent Immunosuppressive Drug Regimen

The renal transplant recipients will receive combination immunosuppressive medications according to SOC at our Institute, with two exceptions. First, MPA (that blocks DNA synthesis in T and B cells) will be initiated 7 days before transplant, at the time of donor DCreg infusion, instead of on the day of transplantation. This is in order to minimize any risk of sensitizing the patient. Historically, pre-treatment of kidney transplant recipients with unmodified donor-specific transfusions and low-dose azathioprine (that acts similarly to MPA) significantly reduced the risk of sensitization (103–107). Furthermore, MPA augments and maintains the regulatory function of DC (108, 109), additionally minimizing any safety concern that DCreg could convert to a stimulatory phenotype after infusion. Second, Ab induction therapy will not be administered at the time of transplant. Patients will be maintained on triple immunosuppressive therapy with MPA, tacrolimus, and prednisone after transplantation, a combination regimen widely applied as SOC at many transplant centers, both in North America and elsewhere worldwide and in The ONE Study of regulatory immune cell therapy in renal transplantation^2^, a trans-Atlantic (European and North American) trial utilizing a unified approach to evaluating immune cell therapy in renal transplantation for the reasons outlined above. The immunosuppressive drug regimen that we propose differs from the regimen (belatacept and rapamycin) that we used together with DCreg in NHP (25). This is because belatacept plus rapamycin is not SOC in human renal transplantation and it is important to assess the safety and efficacy of DCreg in humans in comparison with current SOC, as being evaluated in The ONE study, including the testing of autologous DCreg.

The rationale for not using ATG, alemtuzumab (anti-CD52 mAb) or basiliximab (anti-IL-2Rα mAb) as induction therapy at transplant, is to avoid potential targeting of DCreg infused 7 days before transplant or dampening of immunoregulatory pathways triggered in host T cells by DCreg. In our NHP study, we established that such an approach (pre-transplant immunosuppression at the time of DCreg infusion and avoidance of lymphocyte-depleting induction agents) is both safe and effective. We have opted, however, not to use a Co-B and/or mTOR inhibition-based immunosuppressive regimen, such as that employed in our NHP study, because of the high incidence of acute rejection episodes, including higher grade rejection, in patients receiving Co-B (belatacept), MPA, and steroid therapy and increased side effects in clinical trials of rapamycin-based regimens, either with CNI or MPA (3, 5). Since our initial proposed clinical trial is a *safety* trial, we have chosen to adhere to a safe and proven immunosuppression regimen that does not interfere with DCreg action.

### Persistence of Donor DCreg after Infusion

Monitoring DCreg persistence in the circulation and their tissue homing is essential for understanding their survival and distribution. Flow cytometry techniques to detect donor T cells in peripheral blood of transplant recipients with a threshold sensitivity of one donor cell in 1000 recipient cells (0.1%) are readily available (110). We plan to identify donor DCreg in whole blood at various time points post-transplant, by flow staining for Lin^−^, HLA-DR^+^, BDCA1(CD1c)^+^, CD209 (DC SIGN)^+^, CD11c^+^ DC, in conjunction with staining for a miss-matched donor MHC allele. This approach will allow us distinguish between recipient and donor-derived DCreg. Others (98) have used [^111^In]-oxine to label allogeneic donor-derived myeloid cells (Mreg) for short-term tracking by SPECT imaging following their infusion in renal transplant patients and we will consider using this as a complementary approach.

### Mechanistic and Immunological Monitoring Analyses of Transplant Recipients

Cellular pathways engaged after organ transplantation are complex and involve coordinated interactions between DC as APC and distinct effector and regulatory T cell subsets, which can lead to a state of Ag-cognate effector cell hyporesponsiveness (graft acceptance or quiescence). While it is believed that DCreg are effective in blunting Tmem responses (25, 38–40) and *de novo*-primed naive T cells (13, 34), it is unclear how long after infusion donor-derived DCreg persist in the peripheral blood or in lymphoid tissue of transplant patients, and which mechanism(s) (clonal deletion, anergy, regulation, or exhaustion) may contribute to inducing donor-specific T cell hyporesponsiveness. Our hypothesis is that infusion of donor-derived DCreg (even if their survival is short-lived) (35) will induce donor-specific T cell hyporesponsiveness in the recipient, while nominal T cell recall responses [such as those to anti-Epstein–Barr virus (EBV) or tetanus toxoid (TT)] will be preserved. This could be mediated by decreased donor allo-specific Tmem frequencies and result in residual low allo-specific Tmem proliferation, IFN-γ and Granzyme B/Perforin production in response to donor Ag stimulation, but with preserved responses to EBV and TT stimulation.

To address these questions, we will collect blood samples pre-transplant on day −7 (pre DCreg infusion), on day 0, and at 3 months, 1 year, and 2 year post-transplant. We will (1) characterize the phenotype, memory differentiation, and function of different T cell subsets, (2) assess donor-reactive T cell clonality and function, (3) identify effector and regulatory cells and molecules in for-cause and protocol biopsy samples. While no single immunologic test can identify peripheral hyporesponsiveness after organ transplantation, we will attempt to assess multiple essential T cell immune parameters methodically at the same time, an approach expected to provide a possible signature and mechanism of peripheral anti-donor hyporesponsiveness after DCreg infusion.

We will assess T cell expression of co-stimulatory receptors [e.g., CD28, inducible costimulator (ICOS) and CD40L], which are critical for cross-talk with DC, as well as co-inhibitory receptors [PD-1, TIM3 (T cell immunoglobulin mucin domain 3) and cytotoxic T lymphocyte Ag (CTLA)-4] that are up-regulated on recently activated/exhausted T cells in conjunction with expression of Annexin V/7-AAD to track apoptosis. We will also track EBV-specific and anti-TT T cells as controls for recall responses. We will correlate the levels of memory CD8^+^, CD4^+^ T_FH_, and CD4^+^ Tconv effectors with Treg, DSA titer, plasma cytokines and effector and regulatory cell, IgG, and complement (C4d) deposition in the allograft. Age-matched healthy controls and renal transplant patients who did not receive DCreg infusion will serve as controls.

## Conclusion

There is extensive evidence that DCreg of donor origin can regulate alloimmune responses and promote long-term organ transplant survival in rodents. The recent observation that DCreg can safely prolong renal transplant survival in a robust, pre-clinical NHP model, in which the graft recipients received a minimal immunosuppressive regimen, provides further justification for a clinical trial. Appropriate culture conditions, leading to the manufacture of GMP grade DCreg, which are resistant to maturation and have potential to regulate host alloimmunity, have been developed for clinical testing.

## Author Contributions

AT, AZ, ME, LB, FL, and DM each contributed to the conception and writing of the work, revised it critically, finally approved the submitted version of the manuscript, and agreed to be accountable for all aspects of the accuracy and integrity of the work.

## Conflict of Interest Statement

Angus W. Thomson is co-inventor of a US patent for generation of dendritic cells to enhance transplant tolerance. The remaining co authors declare that the research was conducted in the absence of any commercial or financial relationships that could be construed as a potential conflict of interest.

## Footnotes

^1^https://clinicaltrials.gov

^2^http://www.onestudy.org
